# Editorial: Cellular and molecular regulators in non-neoplastic immune-mediated diseases

**DOI:** 10.3389/fimmu.2025.1694308

**Published:** 2025-09-12

**Authors:** Noha M. Elemam, Iman M. Talaat, Omar A. El Meligy, Jennifer E. Hundt

**Affiliations:** ^1^ Clinical Sciences Department, College of Medicine, University of Sharjah, Sharjah, United Arab Emirates; ^2^ Research Institute for Medical and Health Sciences, University of Sharjah, Sharjah, United Arab Emirates; ^3^ Pathology Department, Faculty of Medicine, Alexandria University, Alexandria, Egypt; ^4^ Pediatric Dentistry and Dental Public Health Department, Faculty of Dentistry, Alexandria University, Alexandria, Egypt; ^5^ Lübeck Institute of Experimental Dermatology, University of Lübeck, Lübeck, Germany

**Keywords:** autoimmune disorders, inflammatory conditions, cellular regulators, molecular signaling pathways, immune-mediated diseases, therapeutic targets

Non-neoplastic immune-mediated diseases, including autoimmune and inflammatory disorders, arise from dysregulated cellular and molecular networks that drive chronic inflammation and tissue damage. Understanding the roles of cellular and molecular regulators is crucial for elucidating the mechanisms that drive these diseases and for developing more targeted and effective therapeutic strategies. In this Research Topic, we aimed to highlight studies that explore the roles of immune cell subsets, cytokines, chemokines, signaling pathways, and genetic factors in disease pathogenesis. By identifying novel immune regulators, the overarching goal is to foster precision medicine approaches that improve diagnosis, treatment, and patient outcomes.

One clinical challenge emerging from such immune dysregulation is chronic, difficult-to-heal skin wounds, particularly those linked to inflammatory disorders. Addressing this, Alghazali et al. investigated the role of Rab7 inhibition in promoting adipose-derived stem cell (ASC) differentiation into keratinocyte-like cells. Treatment with the Rab7 inhibitor CID-1067700 enhanced epidermal marker expression (P63, cytokeratin 5/14, filaggrin), reduced vimentin expression, and increased anti-inflammatory activity. Complementary microarray and protein array analyses revealed upregulation of HMOX-1, downregulation of proinflammatory signaling pathways (TNF, IL-17, chemokine, cytokine-receptor interactions), and reduced cytokine secretion. Together, these results point to the combined regenerative and anti-inflammatory potential of ASCs for managing chronic wounds.

Moving from tissue repair to systemic immune regulation, Wang et al. addressed the challenge of complement activation in autoimmune diseases and transplant rejection. While current complement inhibitors provide systemic blockade but increase the risk of infection, the authors designed bispecific antibodies (bsAbs) that locally recruit endogenous complement regulators, such as factor H (FH) or C4b-binding protein (C4BP), to cell surface antigens. These bsAbs successfully inhibited the classical, lectin, and alternative pathways, thereby protecting erythrocytes, leukocytes, and liposomes from complement-mediated lysis. This innovative approach highlights the potential of targeted complement inhibition to strike a balance between efficacy and safety.

In a different autoimmune context, immune thrombocytopenia (ITP) exemplifies the role of T-cell dysregulation in disease pathogenesis. Its pathogenesis involves both autoantibody production and T-cell-mediated platelet destruction, driven by autoreactive Th1, Th2, and Th17 responses alongside impaired Treg function. Genetic predispositions further exacerbate T-cell abnormalities. The review by Bu et al. emphasized how loss of tolerance underpins these mechanisms and discusses emerging therapies targeting T-cell pathways as promising strategies for ITP management.

Expanding on the theme of systemic autoimmune disorders, Systemic Lupus Erythematosus (SLE) illustrates the interplay between cytokines and organ-specific manifestations. In addition to its characteristic autoantibody production and multi-organ involvement, SLE frequently presents with oral lesions that significantly impair quality of life. Elemam et al. reviewed evidence implicating interleukins, interferons, and growth factors in shaping inflammation, apoptosis, and autoantibody generation. By framing cytokines as central regulators of SLE, the review underscores the therapeutic potential of targeting these pathways to improve both systemic and oral disease outcomes.

Central to these autoimmune processes are lymphocyte subsets, which orchestrate immune homeostasis and defense. Advances in immunophenotyping have refined our understanding of T, B, and natural killer (NK) cell populations, illuminating their developmental trajectories and functional specialization. Dysregulated subset distribution is increasingly recognized in autoimmune diseases, infections, malignancies, and treatment responses. As Chen et al. emphasized, monitoring lymphocyte subsets provides not only mechanistic insights but also valuable diagnostic and prognostic information that can inform precision therapies.

Beyond cellular subsets, molecular mediators also shape immune outcomes. The review by Gao et al. highlighted the role of cathepsin S (CTSS), a lysosomal cysteine protease expressed in immune cells, in regulating antigen presentation, intracellular signaling, and extracellular processes such as protease-activated receptor activation and matrix remodeling. Dysregulated CTSS activity is associated with autoimmune diseases, chronic inflammation, and malignancies, making it a promising therapeutic target for innovative interventions.

Finally, transcriptional regulation offers another layer of immune regulation. An et al. investigated the transcription factor Kruppel-like factor 4 (KLF4), which is known to regulate immunosuppressive and antithrombotic pathways, and its interaction with CD55, a regulator of T- and B-cell responses. Using endothelial cells and macrophages, the authors demonstrated that KLF4 upregulates CD55, which then recruits p-CREB (phosphorylated cAMP-responsive element-binding protein-1) and CBP (CREB-binding protein) to drive KLF4 transcriptional activity. This CD55-KLF4 axis was shown to suppress pro-inflammatory and pro-coagulant proteins while inducing homeostatic factors, revealing a novel mechanism critical for vascular and immune homeostasis.

